# Disease-modifying approach to cardiovascular–kidney–metabolic syndrome: The role of combining GLP-1RAs and SGLT-2is

**DOI:** 10.17925/EE.2026.22.2.5

**Published:** 2026-10-14

**Authors:** Sanja Borozan, Cornelius J Fernandez, ABM Kamrul-Hasan, Sunil Nair, Joseph M Pappachan

**Affiliations:** 1. Department of Endocrinology, Clinical Centre of Montenegro, & Faculty of Medicine, Podgorica, Montenegro; 2. Department of Endocrinology & Metabolism, Pilgrim Hospital, United Lincolnshire Hospitals NHS Trust, Boston, UK; 3. Department of Endocrinology, Mymensingh Medical College, Mymensingh, Bangladesh; 4. Department of Endocrinology, Countess of Chester Hospital NHS Trust, Chester, UK; 5. Faculty of Health, Medicine and Society, The University of Chester, Chester, UK; 6. Faculty of Science, Manchester Metropolitan University, Manchester, UK; 7. Department of Endocrinology, Kasturba Medical College, Manipal, Manipal Academy of Higher Education, Manipal, Karnataka, India

**Keywords:** Cardiovascular disease, cardiovascular-kidney-metabolic (CKM) syndrome, GLP-1RA and SGLT-2i combination therapy, glucagon-like peptide receptor agonists (GLP-1RAs), kidney disease, metabolic disease, obesity, sodium-glucose cotransporter 2 inhibitors (SGLT-2i)

## Abstract

Cardiovascular–kidney–metabolic (CKM) syndrome represents a recent paradigm shift from a traditional, fragmented, disease-specific approach to an interconnected, holistic one driven by shared pathophysiologic pathways that are crucial to the development of multisystem organ dysfunction. The escalating global prevalence of CKM syndrome, associated with industrialization, the obesity pandemic and aging demographics, necessitates a fundamental reframing toward the simultaneous and coordinated management of cardiac, renal and metabolic health. Core to this integrated approach is the development of novel therapeutic options, such as glucagon-like peptide-1 receptor agonists (GLP-1 RA) and sodium-glucose cotransporter-2 inhibitors (SGLT-2i), which target these common mechanisms and have the potential to improve long-term prognosis and reverse trends in cardiovascular mortality. In this article, we suggest a strategic roadmap for CKM syndrome to prioritize early, interdisciplinary intervention focused on addressing modifiable risk factors, weight management and tailored treatment protocols, particularly for high-risk populations, to mitigate the detrimental impact of this multiorgan condition.

Cardiovascular–kidney–metabolic (CKM) syndrome is a term coined to highlight the close associations among type 2 diabetes mellitus (T2DM), obesity, renal disease and cardiovascular disease (CVD) and to enhance an integrated clinical care model for patients. The novel framework proposed by the American Heart Association (AHA) is a paradigm shift in the management of multimorbidity in relation to metabolic syndrome.^1^ As T2DM is the major risk factor for CVD and chronic kidney disease (CKD), and because CVD and CKD increase the risk for each other, such a disease characterization should help to improve management strategies based on the degree of involvement of each system in relation to T2DM. With the introduction of newer antidiabetic medications such as glucagon-like peptide-1 receptor agonists (GLP-1RA) and sodium-glucose cotransporter-2 inhibitors (SGLT-2i) in the early 21st century, a major paradigm shift occurred in the approach to T2DM management, from simple glycemic control to a disease-modifying strategy.^2–4^ Appropriate therapeutic interventions can therefore curtail the risk for CKM syndrome to a great extent in high-risk individuals.

Several studies have explored the utility of newer antidiabetic agents, such as GLP-1RA and SGLT-2i, in the prevention and management of CKM syndrome.^5–8^ However, only few studies are exploring the beneficial effects of combining these new medications to reduce CKM risk and manage established disease.^9,10^ We explore the current evidence on the pathobiology of CKM syndrome, therapeutic strategies for its prevention and management and the potential of combining GLP-1RA and SGLT-2i for CKM prevention and management in this clinical update article.

## CKM syndrome: The historical perspectives

Over the past 50 years, significant global economic growth and increased life expectancy have driven an aging population and a higher prevalence of chronic diseases, creating a major public health challenge. In this context, the interconnections among key non-communicable diseases, such as CVD, kidney disease and T2DM, have gained more attention in response to the growing burden of shared risk factors and on-g oing clinical interactions. Although the term is relatively new, the close interconnection in CKM syndrome was recognized in ancient times. Both Egyptian and Chinese traditional texts documented the importance of the heart–kidney connection.^11^ In 1836, Dr. Robert Bright, in his *Cases and Observations Illustrative of Renal Disease*, described significant structural heart changes in patients with kidney disease.^12^ However, the most significant clinical advances were made in the early 21st century, supported by epidemiological data.^12^

**Figure 1:**
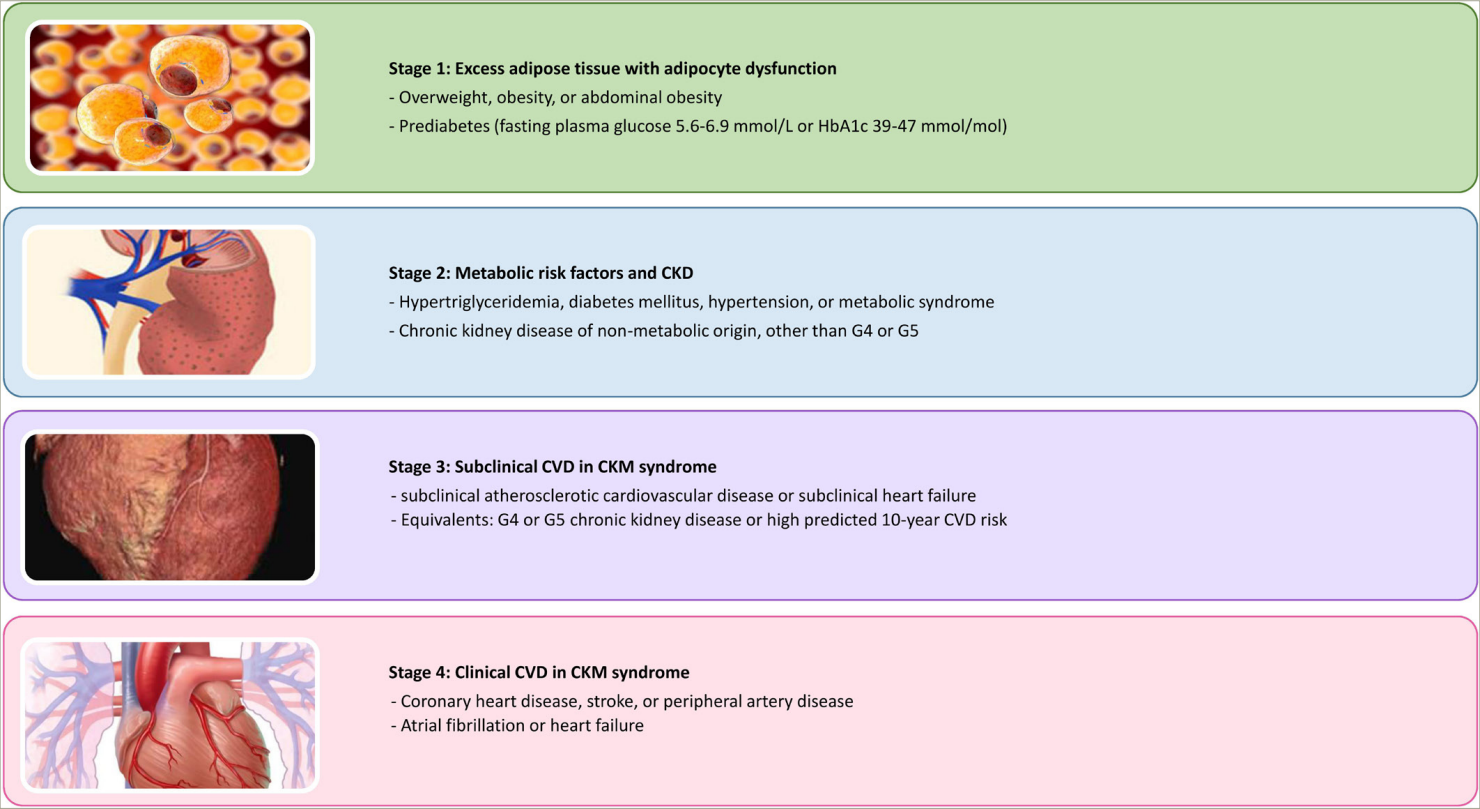
Stages of CKM syndrome

*Figure was made using Microsoft Powerpoint (Microsoft, Redmond, WA, USA)*

*CKD = chronic kidney disease; CKM = cardiovascular-kidney-metabolic; CVD = cardiovascular disease; HbA1c = glycated haemoglobin.*

Recently, the CKM syndrome has been introduced as a framework for a substantial global call to action, advocating for an interdisciplinary approach to address the growing number of patients that modern healthcare systems are facing. In a recent *Presidential Advisory*, the (AHA) established a CKM staging system, in which stage 0 excludes the presence of CKM risk factors; stage I involves excess or dysfunctional adipose tissue (AT) without evidence of subclinical or clinical CKD or CVD; stage II includes the presence of metabolic risk factors (hypertension, T2DM, metabolic syndrome or hypertriglyceridemia) or moderate-to-high-risk CKD; stage III is subclinical CVD in CKM syndrome; and stage IV is clinical CVD.^13^ This staging is depicted in *Figure 1*.^13^

This framework is an attribution to short- and long-term consequences of obesity, recognized as the main risk factor for further metabolic disturbances. At the same time, obesity has already reached pandemic proportions, and recent projections indicate that by 2030, nearly 3 billion adults, approximately 50% of the adult population, will be affected by overweight or obesity.^14^ By that time, 17% of men and 22% of women will be living with obesity, representing an increase of over 115% in 20 years, from 2010 to 2030.^14^ Conversely, despite obesity being one of the most challenging healthcare issues, particularly in middle-income countries, only 7% of countries have established adequate prevention policies.^14^

### Pathobiology of CKM syndrome

The biological basis of CKM syndrome is in the interplay between metabolic, CV and renal derangements, which become the primary drivers of multi-organ damage, increased morbidity and mortality. The proposed central common factor is AT dysfunction, particularly that of visceral and ectopic AT, and consequent alterations in the secretome profile, favoring proinflammatory mediators such as interleukin-6 (IL-6), tumor necrosis factor-alpha (TNF-α) and inflammatory cytokines, which cause low-grade inflammation and oxidative stress. Lower adiponectin levels and triglyceride (TG) accumulation in the liver and skeletal muscle diminish insulin signaling, leading to insulin resistance (IR).^2^ This resistance is a crucial mechanism driving the progression of the syndrome. Recent publications emphasize the emerging role of the gut microbiota in the gut-AT axis in the development of IR and T2DM,^15^ as well as mitochondrial dysfunction as an important contributor to IR, thereby fostering progression from prediabetes to T2DM.^16^

An excess of glucose in the bloodstream activates the local renin–angiotensin–aldosterone system (RAAS) in the myocardium and kidneys, causing vasoconstriction and fibrosis.^5^ In a study by McGavock et al., individuals with impaired glucose tolerance and T2DM had 2.3-fold and 2.1-fold higher myocardial TG content, respectively, compared to lean subjects.^17^ Beyond glucotoxicity, IR is associated with increased circulating free fatty acid (FFA) levels. The resulting ectopic lipid accumulation in the liver, muscle and kidneys causes direct lipotoxic organ damage. Similarly, ectopic fat deposition in the heart leads to cardiac steatosis. These obesity-related myocardial alterations can precede left ventricular dysfunction and heart failure (HF), underscoring the need for an early therapeutic approach.

Endothelial dysfunction in CKM syndrome is one of the most prominent characteristics, driven by inflammatory mediators and metabolic products originating from oxidative stress. Its metabolic consequences include impaired vasodilatation, increased vascular permeability, tissue remodeling and vascular calcifications, as well as a prothrombotic state that further accelerates atherosclerosis, organ hypoperfusion, hypertension and subsequent end-organ damage.^18,19^

As the syndrome advances, multiple molecular mechanisms contribute to kidney involvement, including damage to glomerular, tubular and interstitial structures in a sustained hyperglycemic environment, coupled with overactivity of the RAAS and sympathetic nervous system (SNS).^20^ Podocytes in the kidney glomerulus are insulin-sensitive cells due to the expression of multiple components of the insulin signaling cascade, which regulate glucose transporters (GLUT1, GLUT2, GLUT3, GLUT4 and GLUT8) and enable insulin to modulate their function.^21^ When exposed to hyperinsulinemia, podocytes become insulin-resistant, mostly mediated through degradation of the insulin receptor.^22^ These alterations in podocyte function disrupt homeostasis, leading to further hemodynamic and structural injury, proteinuria and glomerulosclerosis, collectively contributing to a progressive decline in glomerular filtration function. Concurrently, tubular injury driven by chronic glucose and FFA overload secondary to obesity and/or T2DM promotes fibrotic remodeling, with the proximal tubule serving as the predominant site of this metabolic toxicity.^2^

The metabolic dysfunction-associated steatotic liver disease (MASLD) and metabolic-associated steatohepatitis (MASH) have emerged as an important additional amplifier in the advancement of CKM syndrome. Since the primary drivers of CKM syndrome progression, such as glucose, lipids and proinflammatory molecules, are synthesized in the liver, and the liver itself is also a target organ in multi-organ dysfunction, some authors proposed integrating metabolic dysfunction-associated fatty liver disease (MAFLD) as a new component and reframing the condition as the cardiovascular–renal–hepatic–metabolic (CRHM) syndrome.^23,24^ *Figure 2* outlines the pathobiological aspects of CKM syndrome in a nutshell.

### Clinical presentation of CKM syndrome

According to epidemiological data, 90% of the reported population in the USA and the UK met the criteria for CKM syndrome (stages I–IV).^25,26^ Stage II, characterized by metabolic risk factors or kidney dysfunction, was the most prevalent, affecting 49.0% of the U.S. population^25^ and 44.15% of the U.K. population.^26^ In the U.S. population, 15% of adults examined between 2011 and 2020 fulfilled the criteria for more advanced stages III and IV.^25^ Overall, the prevalence of various stages of CKM syndrome differs significantly across age groups, gender and ethnicities.^26,27^ As expected, CKM syndrome stages III and IV are more common in patients older than 60 years, reflecting the cumulative effects of multiple risk factors.^26^ At younger ages, CKM prevalence is higher in males due to the protective properties of estrogen in women during the reproductive period.^25^ Conversely, advanced CKM stages are more frequently seen in postmenopausal women than in men in the same age group.^26^ Reported factors associated with the progression of CKM syndrome include family history of T2DM or CKD, chronic inflammatory diseases and/or elevated highly sensitive C-reactive protein, mental health and sleep disorders, as well as gender-related risk enhancers such as premature menopausal transition, adverse pregnancy outcomes and polycystic ovary syndrome.^27^

**Figure 2:**
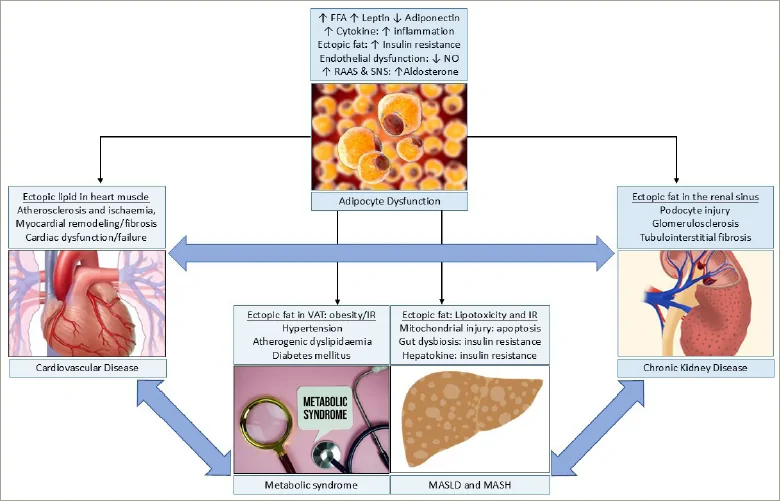
Pathobiological aspects of CKM syndrome

*Adipocyte dysfunction is the central mechanistic link between metabolic syndrome, cardiovascular disease, chronic kidney disease and MASLD/MASH. Figure was made using Microsoft Powerpoint (Microsoft, Redmond, WA, USA)*

*FFA = free fatty acid; IR = insulin resistance; MASLD = metabolic dysfunction-associated fatty liver disease; MASH = metabolic dysfunction-associated steatohepatitis; NO = nitric oxide; RAAS = renin–angiotensin–aldosterone system; SNS = sympathetic nervous system; VAT = visceral adipose tissue.*

The cumulative overlap among the components of CKM syndrome triggers pathophysiological cascades in which the disorder of one system markedly elevates the risk of deterioration in the other two. This risk amplification leads to sequential multi-organ damage and directly contributes to an increased risk of premature mortality. The magnitude of this mortality risk across CKM stages is shown in a recent meta-analysis including 10,330,498 participants.^28^ The analysis revealed a marked escalation in hazard ratios (HR) with advancing stages. Compared to stage 0, individuals in stage IV had markedly elevated risks for all-cause (HR = 3.82), CV (HR = 6.38), coronary heart disease (HR = 9.19),and stroke mortality (HR = 5.48).^26^

Individuals with advanced stages of CKM syndrome face not only a higher burden but also the earlier onset of CVD.^29^ T2DM is often an accelerator and the strongest contributor to CV risk, particularly when coupled with sustained hypertension. Together, they promote kidney damage, including declines in estimated glomerular filtration rate (eGFR) and albuminuria. Conversely, due to their bidirectional relationship, CKD further exacerbates CV risk and HF by worsening hypertension and metabolic abnormalities.^5,13^ Individuals with CKD are more likely to die from a CV event than to progress to terminal renal failure requiring dialysis.^30,31^

In the unidirectional model, clinical outcomes in CKM syndrome reflect the pathogenetic mechanisms involved in multiorgan crosstalk, with almost every vital organ system affected. Beyond major CV events, HF and renal failure, the spectrum of CKM-related consequences includes obstructive sleep apnea syndrome,^20^ cognitive decline and dementia, with a higher odds ratio for male gender and older individuals.^32,33^ A recent study demonstrated that advancing CKM syndrome stages were associated with significant brain structural changes, including reductions in total brain, gray matter and hippocampal volumes, as well as increased white matter hyperintensity volumes.^34^ However, the progression along the CKM trajectory, from stage I to stage IV, is not inevitable. Halting the progression or even achieving a regression to an earlier stage through targeted organ-protective medications (disease-modifying approach) is possible. Indeed, this is the fundamental therapeutic goal of every clinician managing these individuals.

### Management of CKM syndrome

The alarming burden of CKM syndrome worldwide, with a progressive rise of CVD-related and all-cause mortality across its stages,^28,33,35,36^ highly underscores the need for screening of people at risk and timely management.^26,28,33,35–37^ The AHA reinforces a personalized approach for individuals with CKM syndrome, focusing primarily on preventing CV events.^13^ Supporting this strategy, a recent study showed that 45.3% of CV mortality risk is closely associated with advanced stages of CKM syndrome, which could potentially be prevented if the progression of the syndrome is halted and individuals remain in the early stages.^35^

One recently published tool, the PREVENT equation (AHA’s Predicting Risk of CVD Events), estimates short- and long-term total CVD risk in patients with CKM syndrome. This represents an additional step toward preventing end-organ damage, including CVD and HF. The inclusion of eGFR as a predictor and HF as an outcome in the PREVENT equation directly reflects the interplay between CKM components and their synergistic amplification of CV risk.^29^ Another composite indicator is the cardiometabolic index (CMI), which integrates the TG-to-high-density lipoprotein cholesterol ratio (TG/HDL-C) and the waist-to-height ratio. It has been described as a clinically relevant biomarker for CVD risk stratification in individuals with CKM syndrome.^38^

Stage I, defined as the initial manifestation of CKM syndrome, is characterized by dysfunctional adiposity. It is becoming clear that this stage represents a critical step in arresting progression and preventing more serious CV and kidney diseases. Lifestyle interventions, including a healthy diet, regular physical activity and smoking cessation, remain the foundation of the therapeutic approach, as supported by robust evidence from prospective randomized trials and meta-analyses.^39,40^ In a cross-sectional study from five National Health and Nutrition Examination Survey cycles between 2011 and 2020, Li *et al*. showed that intermediate and favourable overall lifestyle scores in adults were associated with 31% and 44% lower prevalence of advanced CKM syndrome stages, respectively.^41^ Furthermore, a systematic article and meta-analysis of 31 randomized controlled trials (RCTs) involving 23,684 participants demonstrated that lifestyle interventions reduce the incidence of T2DM by 41 % and increase the probability of reverting to normoglycemia by 44% in adults with prediabetes.^42^

Nutrition is a key contributor to both optimal health and the development and progression of the CKM syndrome, acting as a modifiable factor across all clinical stages. A balanced and high-quality diet has been linked to a lower risk of all-cause and CV mortality in individuals with CKM syndrome,^43^ highlighting the need to integrate dietary education as a core component of therapeutic strategies. On the other hand, a pro-inflammatory dietary pattern, characterized by higher dietary inflammatory index (DII) scores, is independently associated with greater CKM syndrome severity and increased long-term mortality.^42^ Physical activity is also recognized as an important factor strongly linked to a more favourable CKM stage.^40^ Specifically, high-intensity training is protective against associated CV and metabolic complications.^44^ Engaging in more than 150 min of moderate-to-vigorous physical activity per week is associated with approximately 33% lower odds of CKM stage I, 44% lower odds of stage II, 92 % lower odds of stage III, and 65% lower odds of stage IV.^45^ Smoking cessation is routinely recommended based on its benefits in primary and secondary prevention of major atherosclerotic diseases.^46^ Smoking cessation also helps in preventing other smoking-related adverse health issues.

As CKM syndrome progresses to stage 2 and beyond, pharmacological agents become necessary to achieve adequate control of blood pressure, glucose and lipid levels, thereby mitigating CV risk and interrupting the vicious circle that leads to further CV complications. The traditional pharmacological approach was fragmented and single-diagnosis-centered, involving antihypertensives (angiotensin-converting enzyme inhibitors as first-line agents), alongside statins and, more recently, ezetimibe and proprotein convertase subtilisin/kexin type nine inhibitors (PCSK9i), when needed, to achieve low-density lipoprotein (LDL) cholesterol targets. Similarly, the treatment of T2DM was also historically focused on glucose-lowering drugs, aiming for tight control, which, although effective in reducing microvascular complications, did not sufficiently lower the risk of macrovascular events.^47^ However, the cardiometabolic field has advanced rapidly in recent years, with a growing number of therapeutic options transforming the core approach to treating CKM syndrome. As outlined by the AHA Presidential Advisory in 2023, the new therapeutic framework enables clinicians to alter the natural course of CKM syndrome, foster stage regression and improve clinical outcomes.^13^

### Disease-modifying approach using a combination of GLP-1RAs and SGLT-2is

The introduction of GLP-1RA and SGLT-2i classes of medications challenged the conventional ‘one drug – one organ system – one diagnosis’ paradigm and reshaped the management of T2DM, CVD and CKD. These agents provide benefits that greatly surpass their antihyperglycemic effects. A rationale for combination therapy with these drugs has been recognized due to complementary pathobiological mechanisms that confer additive cardiorenovascular benefits.^48^

GLP-1RAs, such as liraglutide, semaglutide and dulaglutide, are analogs of human GLP-1 secreted by intestinal L-cells. Their mechanisms of action include appetite suppression, reduced food consumption, slower gastric emptying, increased satiety, enhanced glucose-dependent insulin secretion and inhibited glucagon release, resulting in weight loss and improved glycemic control. Additionally, recent studies support the role of GLP-1RA in beta-cell regeneration and in inhibiting apoptosis, thereby preserving beta-c ell mass.^49,50^ However, the rationale for GLP-1RA use from stage I CKM syndrome extends beyond addressing excess AT and other metabolic effects. Large CV outcome trials (CVOTs) consistently demonstrate a significant reduction of major adverse cardiovascular events (MACE) and improvements in kidney function.^51^ Although these agents predominantly reduce fat mass, concomitant reduction in lean body mass has been noted, highlighting the importance of adequate protein intake and resistance exercise.

Aside from their glucose-regulating benefits mediated by promoting urinary glucose excretion, SGLT-2i exhibit pleiotropic effects, including a reduced risk of HF and slowing CKD progression. The well-established antihypertensive class effect was initially attributed to osmotic diuresis; however, recent research indicates that it also involves multiple other mechanisms such as regulation of the RAAS, suppression of the SNS, restoration of ion homeostasis, anti-inflammatory and antioxidant effects, weight loss and improved vascular function.^52^ Consequent reductions in preload and afterload decrease stress on the myocardium, thereby improving cardiac function, mitigating the risk of HF and, in established HF, reducing the frequency of acute episodes and hospitalizations.^53^ Another protective mechanism involves a metabolic shift in cardiac substrate utilization and increased levels of ketones, such as β-hydroxybutyrate and acetoacetate.^54,55^ Potential benefits of elevated ketone levels include increased cardiac energetics and contractile function, prevention of adverse cardiac remodeling and attenuation of the inflammatory profile.^54^

In general, SGLT-2i are preferred in patients with CKD and HF, as they primarily target renal hemodynamics and cardiac metabolism. GLP-1RAs are frequently prioritized for patients with uncontrolled T2DM and/or obesity to mitigate the risk of atherosclerotic CV events due to effects on insulin secretion, weight loss and vascular health. Since the AHA marked obesity as stage I in CKM syndrome, the initial considerations in choosing an appropriate drug should include a favorable effect on body weight (BW), and, notably, both classes of drugs fulfill that criterion. In individuals who progressed to stage II and developed T2DM, combining GLP-1 RA and SGLT-2i corrects seven out of the eight components of the Ominous Octet, with only adipocyte IR remaining unimproved.^48^

Once-weekly semaglutide is the second GLP-1RA approved for weight loss in individuals with overweight or obesity with/without T2DM, following once-daily liraglutide. In the STEP clinical program, treatment with semaglutide resulted in a mean BW change of -15.8% after 68 weeks in individuals without T2DM.^56^ The weight loss is even greater with tirzepatide, a dual glucose-dependent insulinotropic polypeptide (GIP)/GLP-1 agonist. In the SURMOUNT-1 trial, 57% of participants receiving 10 or 15 mg tirzepatide achieved a 20% or greater reduction in BW.^57^ As GLP-1RAs and their combinations with entero-p ancreatic hormones continue to advance, the magnitude of weight loss now approaches that of bariatric surgery, at 25–30%.^58^

By inhibiting glucose reabsorption in the proximal renal tubules, SGLT-2i therapy results in the excretion of approximately 60-80 g of glucose per day, equivalent to 240–320 kcal/day (1 g of glucose equals four calories).^59^ However, the observed weight loss in clinical practice is often lower than expected given glycosuria, likely due to compensatory increases in appetite and food intake, underscoring the complementary role of GLP-1RA effects in appetite suppression.^48^

Large CVOTs have firmly established that both GLP-1RAs and SGLT-2i independently reduce CV and renal risk in T2DM. Emerging evidence, largely from post hoc analyses of RCTs with relatively short-term interventions, observational cohorts and studies focused on surrogate metabolic endpoints, suggests that concomitant use of both drug classes is safe and may provide additional cardiometabolic benefits. However, dedicated large RCTs designed to compare the benefits of combination therapy with either monotherapy for hard cardiovascular or renal outcomes are lacking. While additive benefits appear plausible, the presence of true synergistic effects remains uncertain. Despite the significant proportion of individuals with CKM syndrome requiring comprehensive cardiorenal protection due to multiple risk factors, or already have established CVD and thus will strongly benefit from the use of cardio-reno-protective agents, the concomitant use of GLP-1RA and SGLT-2i in CV outcome trials was rare.^60^

In the AWARD-10 study, adding dulaglutide to an SGLT-2i in patients with inadequately controlled T2DM led to a significant reduction in HbA1c (glycated haemoglobin) and BW.^61^ Similarly, the DURATION-8 trial demonstrated that in poorly controlled T2DM, combination therapy with exenatide and dapagliflozin improved HbA1c, resulting in a weight loss of 5% or more and a reduction in systolic blood pressure (SBP).^62^ An exploratory analysis of the AMPLITUDE-O trial (Effect of Efpeglenatide on Cardiovascular Outcomes) revealed that GLP-1RA benefits on MACE, renal composite outcomes, non-CV death and HF hospitalizations were independent of background SGLT-2i use.^60^

A meta-analysis of RCTs comparing combination therapy with GLP-1RAs and SGLT-2i in elderly patients with T2DM with monotherapy found significant improvements in HbA1c, eGFR, lipid profile and SBP after 6 months, with a favorable safety profile.^63^ In a prospective observational study of individuals with recent myocardial infarction (MI), combination therapy was associated with a significantly lower incidence of MACE compared with either drug alone (*P*=0.009 and *P*=0.008, respectively), independently of HbA1c level.^64^ Based on the data, the authors suggested potential synergistic benefits of combination therapy in improving post-MI remodeling.^64^ A recently published systematic review and meta-analysis of 17 RCTs comparing monotherapy with either agent alone and combination therapy in patients with T2DM patients with established CVD or high CVD risk observed that the combination therapy was associated with comparable CV benefits to either drug alone for 3-point MACE and HF hospitalization. Evidence remains insufficient to claim pharmacological synergy, as studies were not designed to directly test synergistic effects.^65^

**Table 1:** Mechanistic and clinical effects of GLP-1RAs, SGLT-2is and their combination^51,57,66–96^

Outcome	GLP-1RA	SGLT-2i	Combination	Dominant	Key evidence
Body weight	↓ 5–15%^57,66^	↓ 2–4%^77,78^	↓ 8–20%^62,96^	GLP-1RA	STEP, SUSTAIN and DURATION-8
HbA1c	↓ 1–2%^67,68^	↓ 0.5–1%^77–80^	↓ 1.5–3%^61,62,96^	GLP-1RA	LEADER, SUSTAIN-6 and EMPA-REG
Albuminuria	↓ 20–35%^67,69,70^	↓ 30–40%^81–84^	↓ 40–60%^69,70,96^	SGLT-2i	CREDENCE, DAPA-CKD and LEADER
CKD progression	↓ 18–24%^70^	↓ 30–40%^81,83,86,87^	↓ 40–50%^70,81^	SGLT-2i	CREDENCE, DAPA-CKD and EMPA-KIDNEY
HF hospitalization	↓ 9–11%^51,71^	↓ 25–35%^77–80,85^	↓ 35–45%^62,96^	SGLT-2i	EMPA-REG, CANVAS and meta-analysis
3-point MACE	↓ 12–15%^68,69,72^	↓ 10–14%^77–80,85^	↓ 20–30%^51,80^	GLP-1RA	LEADER, SUSTAIN-6 and REWIND
CV death	↓ 12–13%^73^	↓ 14–18%^77–80,85^	↓ 20–30%^51,80^	SGLT-2i	EMPA-REG and meta-analysis
CRP/inflammation	↓ 39–48%^74^	↓ 10–25%^88,89^	↓ 30–50%^74,88^	GLP-1RA	STEP, SUSTAIN and EMPA-REG
Liver fat (MRI-PDFF)	↓ 30–50%^75^	↓ 15–30%^90–92^	↓ 40–60%^75,90–92^	GLP-1RA	STEP 1, E-LIFT, LEAD and meta-analysis
Visceral AT	↓ 20–30%^76^	↓ 5–10%^93,94^	↓ 15–30%^62,96^	GLP-1RA	STEP, DURATION-8 and meta-analysis
Systolic BP	↓ 2–6 mmHg^67–69^	↓ 3–5 mmHg^77–79,95^	↓ 5–10 mmHg^62,96^	SGLT-2i	EMPA-REG and DURATION-8

*AT = adipose tissue ; BP = blood pressure; CKD = chronic kidney disease; CRP = C-reactive protein; CV = cardiovascular; GLP-1RA = glucagon-like peptide-1 receptor agonists ; HbA1c = glycated haemoglobin; HF = heart failure; MACE = major adverse cardiovascular events; MRI-PDFF = magnetic resonance imaging-proton density fat fraction; SGLT-2i = sodium-glucose cotransporter-2 inhibitors.*

Despite their established benefits, the GLP-1RAs and SGLT-2i have important limitations, including high cost, restricted access, adherence challenges, gastrointestinal side effects, euglycemic diabetic ketoacidosis and potential lean body mass loss accompanying weight loss. Additional caution is warranted in frail older adults and in patients with advanced CKD, emphasizing the need for individualized treatment options.

A summary of the mechanistic and clinical effects of GLP-1RA, SGLT-2i and their combination is given in *Table 1*.^51,57,66–96^ A summary of the available evidence from the RCTs, meta-analyses and imaging studies on GLP-1RA and SGLT-2i-based combination therapy is given in *Table 2*.^61,62,75,96,97^ A summary of mechanistic rationale and clinical implications of combination therapy is given in *Table 3*.^65^ An illustration of therapeutic targets and clinical benefits of GLP-1RA, SGLT-2i and their combination is given in *Figure 3*. A mechanistic illustration of complementary effects of GLP-1RA and SGLT-2i in CKM syndrome is given in *Figure 4*. Finally, practical CKM management algorithm stratified according to disease stage and predominant organ involvement is illustrated in *Figure 5*.

### Areas of uncertainty

Although an increasing amount of data on hemodynamic, metabolic and other benefits is currently available, definitive long-term effects of combination therapy still need to be proven in larger RCTs. Several on-going studies are investigating the benefits of combination therapy. For instance, a 32-week randomized, double-blind, placebo-controlled trial (RESILIENT) is designed to assess the effects of co-administering exenatide and dapagliflozin on energy balance, total fat mass, regional fat mass and organ-specific fat mass measured by dual-energy X-ray absorptiometry (DXA) and multiorgan insulin sensitivity in obese patients with T2DM.^98^

New therapies for CKM syndrome are in the pipeline. Triple agonists (GLP-1/GIP/glucagon) such as retatrutide, lysophosphatidic acid receptor antagonists such as bexotegrast and second-generation mineralocorticoid receptor antagonists such as esaxerenone and balcinrenone have significant potential to transform the pathophysiological trajectory of CKM syndrome.^20^ Whether these agents, with their anti-inflammatory and antifibrotic properties, will complement existing therapies remains an open question. Although dedicated trials in CKM syndrome are lacking, retatrutide reduces BW 24%, liver fat 82%, with reduction of BP and lipids suggesting potential use.^99^

**Table 2:** RCTs, meta-analyses and mechanistic/imaging studies on GLP-1RA and SGLT-2i combination therapy^61,62,75,96,97^

Study	Design	Population	Sample	Duration	End points	Major findings	Limitations
DURATION-8^62^	Multicenter double-blind phase 3 RCT. Exenatide, dapagliflozin or a combination	T2DM on metformin	n=695	28 weeks	HbA1c (primary), weight, BP	HbA1c ↓ 2.0%; weight ↓ 3.4 kg; SBP ↓ 4–6 mmHg; greater glycemic, BP and weight effect	Short duration; no CV or renal outcomes; low-risk population
AWARD-10^61^	Multicentre double-blind phase 3b RCT. Placebo versus dulaglutide 0.75 versus dulaglutide 1.5 mg	T2DM on SGLT-2i (with or without metformin)	n=424	24 weeks	HbA1c (primary), safety	HbA1c ↓ 1.3–1.5%; weight ↓ 2–3 kg; additive glycemic control; acceptable tolerability/safety	Short duration; no CV or renal outcomes; no GLP-1RA alone arm for comparison
SUSTAIN-9^96^	Multicentre double-blind phase 3 RCT. Placebo SC OW versus semaglutide 1.0 mg SC OW	T2DM on SGLT-2i (with or without metformin)	n=302	30 weeks	HbA1c (primary), weight, safety	HbA1c ↓ 1.4% additional; weight ↓ by 3.8 kg; SBP ↓ by 3 mmHg; well tolerated	Short duration; no CV or renal outcomes; low-risk population
Meta-analysis by Li et al. (2022)^97^	Systematic article and meta-analysis of RCTs	T2DM (mono or combo therapy)	n=1,895	12–104 weeks	HbA1c (primary), weight, BP, safety	HbA1c ↓ 0.6–0.9% versus mono; weight ↓ by 1.5–3 kg; SBP ↓ by 2–4 mmHg (extra effect)	Small size; short duration; heterogeneous design
STEP MASLD^75^	Small RCTs and sub-studies	Obesity ± T2 DM	n=50–500	12–68 weeks	VAT, liver fat (MRI-PDFF), CRP	VAT ↓ 15–30%; liver fat ↓ 40–60%; CRP ↓ 30–50% (variable)	Small sample sizes; surrogate endpoints only

*BP = blood pressure; CRP = C-reactive protein; CV = cardiovascular disease ; HbA1c = glycated haemoglobin; MASLD = metabolic dysfunction-associated steatotic liver disease; MRI-PDFF = magnetic resonance imaging-proton density fat fraction; PDFF = proton density fat fraction; PDFF = proton density fat fraction; RCT = randomized control trial; SBP = systolic blood pressure; SGLT-2i = sodium-glucose cotransporter-2 inhibitors; T2DM = type 2 diabetes mellitus; VAT = visceral adipose tissue.*

**Table 3:** Mechanistic rationale and clinical implications of combination therapy^65^

Domain	Key points	Clinical implications
Mechanistic rationale	GLP-1RAs primarily ↓ appetite, body weight, visceral adiposity, hepatic steatosis, inflammation and ASCVD risk. SGLT-2i primarily ↓ intraglomerular pressure, plasma volume, blood pressure, HF risk and CKD progression	Mechanisms are complementary; hence, dual therapy produces an additive effect and offers greater risk reduction than either agent alone
Additive versus synergistic effects^65^	Predominantly additive rather than synergistic effects in ↓ body weight, HbA1c, BP and albuminuria with dual therapy. A recent meta-analysis^65^ ruled out a synergistic amplification of MACE, HF hospitalization, or other cardiovascular outcomes	Combination (dual) therapy is predominantly complementary, with additive rather than synergistic benefits on cardiorenal outcomes
Body weight and ASCVD	GLP-1RA is dominant; SGLT-2i provides modest reduction	For obesity, MASLD/MASH and CKM
Cardiorenal outcomes	SGLT-2i is dominant for HF, albuminuria, CKD and CV death; GLP-1RA gives an additional ↓ ASCVD events and inflammation	Dual therapy offers greater cardiorenal effects in high-risk T2DM
Patient selection	Obesity, T2DM, established ASCVD, CKD, HF, albuminuria, MASLD/MASH or multiple cardiometabolic risk factors	Dual therapy is optimal for those with components of CKM syndrome
Current limitations	No large dedicated RCTs with dual therapy. Most ASCVD and cardiorenal benefits are extrapolated from individual RCTs	Additive cardiorenal effects require confirmation from dedicated RCTs
Uncertainties	Sequential versus simultaneous initiation? universal dual therapy?	Timing of dual therapy initiation is vital
Future strategies	Dual therapy: ideal for those with obesity, ASCVD, HF, and CKD	Evidence to be available and affordable

Validated biomarkers that integrate oxidative and endothelial activities remain an unmet need in CKM syndrome. Potential candidates like adipokines and vascular modulators such as retinol-binding protein 4 (RBP4), lipocalin 2 (LCN2), apolipoprotein M (ApoM), Klotho and matrix Gla protein (MGP) still require extensive validation and standardized cut-off values.^18^ Advances in clinical diagnosis and monitoring may enable early detection and phenotyping of CKM syndrome, potentially leading to precision strategies to disrupt the self-reinforcing cardiorenal damage.

A new field exploring the modulatory effects of the gut microbiota on gut-AT axis alterations and consequent low-grade inflammation has provided new insights into the factors driving the development and progression of CKM syndrome. A cross-sectional study by Yan et al. linked a high dietary index for gut microbiota (DI-GM ≥6) to a 25% reduction in the risk of advanced CKM, highlighting the protective role of an anti-inflammatory gut microbiota profile.^100^

## Conclusion

Moving beyond a prolonged period of fragmented, diagnosis-specific patient care, AHA finally introduced a new framework underscoring the necessity of a holistic approach. This framework emphasizes that the heart, kidneys and metabolism must be viewed and treated simultaneously. This shift comes at a time when, globally, the prevalence of CKM syndrome continues to rise in line with industrialization, the obesity pandemic and demographic aging. The interconnected and complex nature of metabolic disorders, CV disease and CKD in CKM syndrome demands a coordinated framework targeting the core pathophysiological mechanisms, such as IR, low-grade inflammation and endothelial dysfunction. Thus, an integrated approach to prevention, early detection and timely, interdisciplinary management aimed at promoting weight loss, improving metabolic control and addressing CVD risk factors has emerged as a goal to markedly reduce the consequences of CKM syndrome.

**Figure 3:**
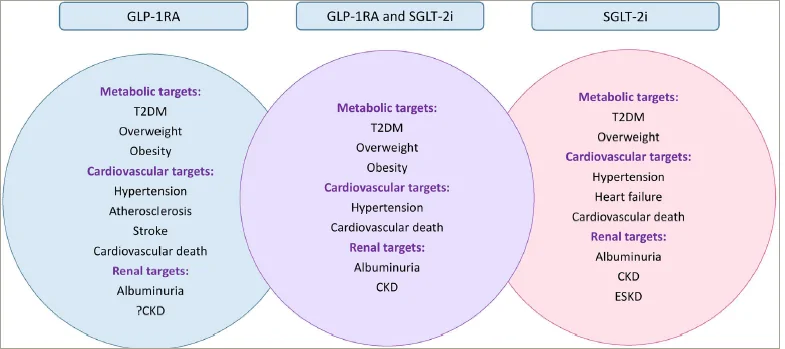
Therapeutic targets and clinical benefits of GLP-1RA, SGLT-2i and their combination

*GLP-1RAs demonstrate particular benefits in reducing ASCVD, whereas SGLT-2i demonstrates particular benefits in HF, CV death and CKD progression. The overlapping section highlights the shared therapeutic targets. Figure was made using Microsoft Powerpoint (Microsoft, Redmond, WA, USA) ASCVD = atherosclerotic cardiovascular disease; CKD = chronic kidney disease; CV = cardiovascular; ESKD = established kidney disease; GLP-1RA = glucagon-like peptide-1 receptor agonists; HF = heart failure; SGLT-2i = sodium-glucose cotransporter 2 inhibitors; T2DM = type 2 diabetes mellitus.*

**Figure 4:**
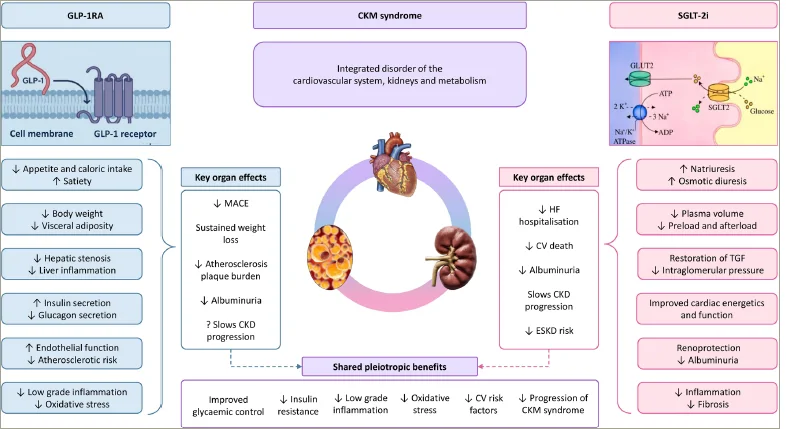
Complementary effects of GLP-1RA and SGLT-2i in CKM syndrome

*A mechanistic illustration of complementary effects of GLP-1RA and SGLT-2i in CKM syndrome. In subjects with CKM syndrome, GLP-1RAs reduce the risk of MACE, achieve sustained weight loss, reduce atherosclerotic plaque burden, reduce albuminuria and possibly slow CKD progression, whereas SGLT-2i reduces albuminuria, slows CKD progression, reduces ESKD risk, lowers the HF hospitalisation and reduces cardiovascular death*

*ADP = adenosine diphosphate; ATP = adenosine triphosphate; CKD = chronic kidney disease; CKM syndrome = cardiovascular–kidney–metabolic syndrome; CV = cardiovascular; ESKD = established kidney disease; GLP-1RA = glucagon-like peptide-1 receptor agonists; GLUT2 = glucose transporter 2; HF = heart failure; MACE = major adverse cardiovascular events; TGF = tubuloglomerular feedback.*

**Figure 5:**
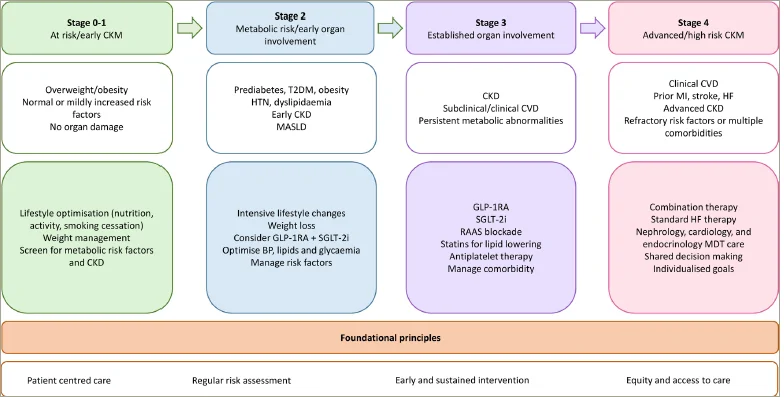
A practical management algorithm for CKM syndrome

*A practical management algorithm for CKM syndrome stratified according to disease stage and predominant organ involvement. Figure was made using Microsoft Powerpoint (Microsoft, Redmond, WA, USA)*

*BP = blood pressure; CKD = chronic kidney disease; CKM syndrome = cardiovascular–kidney–metabolic syndrome; CVD = cardiovascular disease; GLP-1RA = glucagon-like peptide-1 receptor agonists; HF = heart failure; HTN = hypertension; MASLD = metabolic dysfunction-associated steatotic liver disease; MDT = multidisciplinary team; MI = myocardial infarction; RAAS = renin–angiotensin–aldosterone system; T2DM = type 2 diabetes mellitus.*

The notable pharmacotherapeutic advances in the last two decades in the field of obesity and related complications with novel agents such as GLP-1RA and SGLT-2i have the potential to reshape the long-term prognosis of CKM syndrome and reverse the unidirectional trend toward CV mortality. While the optimal therapeutic approach across populations is still being determined, a strategic roadmap should prioritize early intervention, particularly for high-risk individuals. Based on current evidence, the combination of GLP-1RAs and SGLT-2i appears to be a promising therapeutic option for CKM syndrome; however, further dedicated RCTs are needed to confirm its incremental benefit over monotherapy and potential synergistic effects.

### Article Highlights

- CKM syndrome is a term coined to highlight the close associations between T2DM, obesity, renal disease and CVD to enhance an integrated clinical care model for patients.
- Endothelial dysfunction driven by inflammatory mediators and oxidative stress results in impaired vasodilatation, increased vascular permeability, tissue remodeling, vascular calcifications and a prothrombotic state that further accelerates atherosclerosis, organ hypoperfusion, hypertension and subsequent end-organ damage.
- The recently published tool by the AHA, the PREVENT equation, helps estimate the short-term and long-term CVD risks in patients with CKM syndrome.
- Newer antidiabetic agents with disease-modifying properties in patients with T2DM and obesity, such as GLP-1RAs and SGLT-2i, can also modify the clinical outcomes in CKM syndrome.
- The combination therapy with GLP-1RA and SGLT-2i appears to possess additive effects in managing patients with CKM syndrome, an emerging concept that needs more research to develop clinical practice recommendations.
